# Medical History from the Earliest Times

**Published:** 1893-02-18

**Authors:** E. T. Withington


### Medical History from the Earliest Times.

By E. T. Withington, M.A., M.B. Oxon.

### XLIII.—THE MEDICAL PROFESSION IN THE MIDDLE AGES.

"Whosoever will henceforth practise medicine, let him present himself to our officials and judges to be examined by them; but if he presume of his own temerity, let him be imprisoned and all his goods be sold by auction. The object of this is to prevent the subjects of our kingdom incurring peril through the ignorance of physicians."

The above edict, promulgated in 1140 by Roger II., King of the two Sicilies, is the first known legal enactment for the regulation of medical practice in Europe. Roger, who was a great friend of "Saracenic studies," had many Arabs at his court, and his medical law was not improbably copied from the regulations long in force in the East. His grandson, the Emperor Frederick II., called the "World's Wonder," was notorious for his addiction to Moslem manners and learning, and his medical enactments are of great interest and importance. The following are the chief: "Considering the harm which may arise from the ignorance of physicians, we ordain that no one shall henceforth practise physic unless he be first publicly examined by the masters at Salerno, and present testimonials, both from them and from those appointed by us, to ourselves or our representative, and receive from us or him licence to practise." "Since the science of medicine can never be understood without some previous knowledge of logic, we decree that none shall study medicine unless he have studied logic for at least three years. Then let him learn medicine for five years, and also surgery, which is a part of medicine." On receiving his licence he must take oath to attend the poor gratis, and to denounce any frauds of the apothecaries, and must still remain for a year under the supervision of some older practitioner. "He shall visit the sick at least twice daily, and once at night, if required. He shall not charge a patient in the town more than half a tarenus, or one in the country more than three tareni, if his expenses are paid, or four if he pays his own"—a "tarenus" was worth about three shillings. "No surgeon shall practise until he has given evidence to the masters of the medical faculty of having studied that part of medicine at least a year, and especially of having thoroughly learnt human anatomy, without which neither can incisions be safely made, nor fractures cured."

The sale of poisons was simply and effectually regulated. "Whosoever shall have or sell any poison or noxious drug, not useful or necessary to his art, let him be hanged." "Those who give magic drinks and love philtres, if they cause bodily harm, shall be punished with death, but if the recipient is uninjured, they shall forfeit all their goods and suffer a year's imprisonment. Investigators of truth and nature know that these arts are foolish and fabulous, but even the intent to do evil should not go unpunished." "We consider it our duty," continues the scientific Emperor, "to preserve, as far as possible, the salubrity of the air; wherefore we decree that no hemp or flax be placed for maturing in water within a mile of any town or camp, for we have learnt for certain that the air is corrupted thereby; let those who do so forfeit their hemp or flax and be brought before our courts. We order that the depth of graves be not less than half a 'canna'; penalty, a fine of one augustale." Bodies of animals and other refuse must be suitably disposed of on pain of a fine of one augustale for things larger than a dog, and half that sum if smaller.

Among university studies medicine ranked lowest of the superior faculties, but as in ancient times it might boast of being the only profession which had produced a god, so in the middle ages the beneficent science gained for its students the beautiful title "ordo gratiosus." The title "doctor" was given even in classical times to teachers of the liberal arts, but it was first employed in something like its modern sense at the end of the twelfth century by Gilles of Corbeil, who uses it to denote the Salernitan masters, while about the same time Roger of Parma calls his teacher "noster doctor," "egregius doctor." With the addition of various complimentary epithets, the term was applied to the great schoolmen of the thirteenth century, and Arnald of Villanova calls himself indiscriminately "doctor" and "magister" medicinae. By the end of that century it had become a recognised degree at Salerno, whence it spread to other universities. The candidate, after going through the above-mentioned curriculum, was further required to defend four theses on passages from Hippocrates, Aristotle, Galen, and one modern author. He then took oath not to contradict the college; to teach nothing false; to recommend the sacrament of confession to his patients; to receive no pay from the poor, even when offered; to enter into no dishonest league with apothecaries; and to administer no noxious drug. Finally, he received a ring, a wreath of laurel and ivy, a book first closed and then opened, the kiss of peace, the rank of Doctor in Philosophy and Medicine, "et facultatem has scientias ubique terrarum profitendi, exercendi, docendi, interpretandi, corrigendi, et de iis quae ad illas spectant disserendi, necnon quod visum fuerit sua auctoritate statuendi, ac ubilibet cathedram ascendendi," but he must not practice surgery on pain of a fine of 500 ducats.

Mediæval surgeons may be roughly divided into three classes: (1) those of the "long robe," such as Lanfranc and Guy of Chauliac, who equalled the physicians in learning, and usually surpassed them in practical knowledge; (2) the bulk of the profession who were closely connected with the barbers and bath-keepers; and (3) the wandering lithotomists, rupture curers, and other specialists. The two latter classes were much despised by the "pure physicians," but we shall shortly come across representatives of both, who are justly famous in medical history. A description of the relations of these several grades to one another, and of the regulations concerning them in various countries, would far exceed the scope of this article, and we need only mention that they were sometimes licensed by the bishops, a practice originating perhaps in the custom, of which an example has already been given, of obtaining their permission before performing any perilous operation.

One of the earliest signs of the revival of medicine was the restoration of the public or communal physicians, as at Bologna and Venice, the mercantile people of which latter city, with a keen eye for obtaining the best value for their money, did not hesitate to employ even unconverted Saracens. In 1436 the Emperor Sigismund decreed that every imperial city in Germany should have a public medical officer, who should be paid 100 gulden yearly from Church revenues, and attend the poor gratis, for the "high masters in physic never do this, and therefore they go to hell." The "archiatri of the palace," or royal physicians, were restored in the reign of the Emperor Charles the Bald, whose Jewish doctor, Zedekiah, was accused, probably with equal truth, of being a sorcerer and of poisoning his master. It is in this capacity that we meet with the last Greek practitioner who might trace some connection with the great men of old. Angelus of Cyprus, for seven years (1364-71) physician to Charles the Bad of Navarre, was "a very learned clerk, versed in the Latin tongue, and powerful in argument"; so his master suggested that he should use these qualities to ingratiate himself with his cousin, Charles V., of France, and take an early opportunity of poisoning that monarch. Rather than obey, the physician fled from the court, and was never seen again, but report said that he had been overtaken and put to death by the king's order.

